# Potentiation of a Porous Silicon Therapeutic Vaccine in Colorectal Cancer via Oxaliplatin-Mediated Regulation of Myeloid-Driven Immunosuppression

**DOI:** 10.3390/jfb17040185

**Published:** 2026-04-10

**Authors:** Yongbin Liu, Busra Akay Hacan, Junjun Zheng, Xueying Ge, Dongfang Yu, Zhe Chen, Yitian Xu, Ning Shao, Haifa Shen, Xuewu Liu, Roderic I. Pettigrew, Ping-Ying Pan, Shu-Hsia Chen, Junhua Mai

**Affiliations:** 1 Center for BioNanoengineering, Houston Methodist Academic Institute, Houston, TX 77030, USA; 2 Center for Immunotherapy and Neal Cancer Center, Houston Methodist Academic Institute, Houston, TX 77030, USA; 3 Immunomonitoring Core, Houston Methodist Neal Cancer Center, Houston, TX 77030, USA; 4 School of Engineering Medicine/ENMED, Texas A&M University and Houston Methodist Hospital, Houston, TX 77030, USA

**Keywords:** oxaliplatin, cancer vaccine, myeloid-derived suppressor cells (MDSCs), colorectal cancer (CRC)

## Abstract

Although immunotherapy has shown great promise in treating various types of cancer, advanced tumors are often refractory due to a highly immunosuppressive tumor microenvironment (TME). We previously engineered a cancer therapeutic vaccine platform, µGCVax, by co-loading tumor antigen peptides, STING and TLR9 agonists into porous silicon microparticles. While effective in models with lower disease burden, its efficacy against advanced colorectal cancer (CRC) was less promising due to the accumulation of myeloid-derived suppressor cells (MDSCs) in TMEs. In this study, we investigated whether µGCVax-based immunotherapy in advanced CRCs could be potentiated via regulating MDSCs to reprogram the TME. In an advanced CT26 murine CRC model, we assessed µGCVax in combination with oxaliplatin, a standard CRC chemotherapeutic with established immunomodulatory effects. We demonstrated that oxaliplatin was preferentially taken up by monocytic MDSCs (M-MDSCs) and effectively reduced their abundance in the bone marrow, blood, spleen, and tumor. Relief of this immunosuppressive TME increased intratumoral infiltration of antigen-specific CD8^+^ T cells. Ultimately, the combination of oxaliplatin with µGCVax induced robust regression of established CRC tumors. These findings highlight that oxaliplatin synergizes with µGCVax by overcoming MDSC-mediated immunosuppression and enhancing antitumor immunity, representing a promising chemo-immunotherapy strategy for advanced CRC.

## 1. Introduction

Colorectal cancer (CRC) is one of the most common malignancies worldwide and represents the second leading cause of cancer-related mortality each year [1,2]. A substantial proportion of patients are diagnosed with advanced or metastatic CRC, and the incidence of CRC has continued to rise in recent years [3,4]. Despite the availability of multiple therapeutic options, the overall prognosis for patients with CRC remains unsatisfactory. In recent years, cancer immunotherapies, such as immune checkpoint inhibitors and therapeutic cancer vaccines, have been explored as novel treatment strategies for CRC, aiming to eliminate tumor cells by activating the host immune system [5,6,7,8,9,10,11]. However, the clinical benefits of current immunotherapeutic approaches have been largely confined to patients with microsatellite instability-high (MSI-H) CRC, while patients with microsatellite-stable (MSS) CRC generally fail to mount effective therapeutic responses [12,13,14]. With early therapeutic cancer vaccines, although robust cancer-specific immunity was stimulated post-vaccination, overall treatment outcomes are still suboptimal.

Previously, we reported a therapeutic vaccine in which the STING agonist 2′3′-cGAMP, TLR9 ligand CpG, and tumor antigen peptides were co-loaded into the nanopores of porous silicon microparticles (µGCVax) [15,16]. The nanoporous silicon microparticles facilitate the coordinated delivery of tumor antigen peptides and innate immune agonists into antigen-presenting cells, thereby enhancing cross-presentation and cytotoxic T-cell priming. This µGCVax-based therapeutic vaccine robustly activated cytotoxic T-cell responses and elicited potent antitumor efficacy in murine models of early-stage melanoma, breast cancer and CRC. Despite its therapeutic efficacy, tumor recurrence and therapeutic resistance have been observed, particularly in large, established tumors. Therefore, the impact of the therapeutic vaccines on advanced-stage disease requires further investigation. In addition, combination with other therapeutic modalities may be necessary to enhance antitumor efficacy in large and resistant tumors.

One major factor of the failure of cancer immunotherapy is the immunosuppressive tumor microenvironment (TME), in which myeloid-derived suppressor cells (MDSCs) play a key role in limiting therapeutic efficacy [17,18]. MDSCs include monocytic MDSC (M-MDSCs) and polymorphonuclear MDSCs (PMN-MDSCs), both of which have strong immunosuppressive activity [19,20]. MDSCs inhibit antitumor T-cell responses within TMEs through multiple mechanisms, including production of reactive oxygen/nitrogen species (ROS/RNS), metabolism starvation, secretion of immunosuppressive enzymes and soluble factors, impairment of antigen presentation, and direct inhibitory ligand–receptors interactions with T cells [21,22]. Therefore, MDSCs can be a therapeutic target to alleviate their immunosuppressive activities and enhance the T-cell response and antitumor efficacy [23].

Oxaliplatin is a platinum-based chemotherapy drug that binds to DNA, resulting in the apoptosis of cancer cells [24,25]. Oxaliplatin-based chemotherapy remains as a first-line treatment in CRC [26,27]. In addition to its direct cytotoxic effect on cancer cells, oxaliplatin induced immunogenic cell death (ICD) [28] and enhanced T-cell populations [29,30,31]. Moreover, it effectively reprograms the TME of CRC by both reducing MDSC abundance and attenuating these cells’ immunosuppressive function [31,32]. Oxaliplatin has been used in combination with immune checkpoint inhibitors-based cancer immunotherapy, which successfully suppressed tumor growth [33]. Thus, we reasoned that combining µGCVax (to amplify antigen-specific CD8^+^ T-cell priming) with oxaliplatin (to induce ICD and reduce immunosuppressive MDSCs) would overcome key barriers in advanced MSS CRC.

In this study, we investigated the combination of µGCVax and oxaliplatin in an advanced CRC model with MSS features. The therapy synergistically enhanced antitumor efficacy by promoting antigen-specific CD8^+^ T-cell infiltration and cytotoxicity while reducing MDSCs, particularly M-MDSCs. Oxaliplatin selectively depleted M-MDSCs in vivo and in vitro, suggesting that this combination represents a promising strategy for treating advanced CRC.

## 2. Materials and Methods

### 2.1. Cell Lines

Murine CRC cell line CT26 was purchased from American Type Culture Collection. Cells were cultured in Dulbecco’s Modified Eagle’s Medium (DMEM, Corning, NY, USA) supplemented with 10% fetal bovine serum (FBS, GenDEPOT, Katy, TX, USA) and penicillin and streptomycin (1: 100) (GenDEPOT) at 37 °C in 5% CO_2_.

### 2.2. Preparation of GP70-Loaded µGCVax (µGCGP70)

µGCVax and liposomes encapsulated with soluble adjuvants and antigens were prepared as described [15,16,34]. In brief, for each mouse, soluble adjuvants (10 μg CpG, 5 μg 2′3′-cGAMP, Invivogen, San Diego, CA, USA) and GP70 peptide (SPSYVYHQF, 100 μg, Peptide 2.0, Chantilly, VA, USA) were dissolved in sterile water and mixed with 0.1% Tween-20, 20 mg/mL 1,2-dioleoyl-sn-glycero-3-phosphocholine and t-butanol. Subsequently, samples were freeze-dried. Liposomes were reconstituted by adding sterile water into the powder and then loaded into 0.6 billion 1 μm porous silicon particles via brief sonication. Antigen and adjuvant contents in µGCVax were monitored as described using Cy5-labeled CpG [15]. After loading, µGCVax particles were spun down at 21,000× *g* for 10 min to collect supernatant, and fluorescent intensity was measured at Ex 630 nm/Em 671 nm.

### 2.3. Animal Studies

Female BALB/c mice (6–8 weeks old) were purchased from Jackson Laboratory (Bar Harbor, ME, USA). All animal studies were performed in accordance with the protocols (IS00007302) approved by the Institutional Animal Care and Use Committee (IACUC) at the Houston Methodist Research Institute.

To generate the CRC-bearing mouse model, 1 × 10^6^ CT26 cells were subcutaneously injected into the right flank of BALB/c mice. Mice were then treated with µGCGP70 vaccine 3 or 7 days after tumor inoculation when tumor volume reached approximately 50 mm^3^ or 200 mm^3^, respectively, to monitor therapeutic outcomes. Tumor volume was calculated as 0.5 × L × W^2^ (L, length; W, width).

For the combination therapy study, CT26 tumor-bearing mice received 7 mg/kg oxaliplatin via intravenous injection (i.v.) and µGCGP70 via footpad injection [15], as we previously reported. Oxaliplatin was injected every 3 days for 3 total injections and µGCGP70 was injected twice every 7 days. Tumor size was measured by calipers every 2 days.

To account for potential effects of tumor size on immune cell analysis, size-matched tumors [35] were selected across all experimental groups. Accordingly, tumor cells were inoculated 4 days earlier in the combination therapy group and 2 days earlier in the monotherapy groups relative to the control group to ensure comparable tumor sizes at the start of treatment, while the treatment duration and dosing regimen remained consistent across all groups.

### 2.4. Cell Isolation

The fresh tumor tissues were cut into pieces and digested in RPMI 1640 (Corning) with collagenase IV (Worthington-Biochem, Lakewood, NJ, USA) and DNase (Fisher Scientific, Waltham, MA, USA) for 1 h. Cell suspensions were filtered through Nylon cell strainers (40 μm, Bio basic, Markham, ON, Canada), and red blood cells were lysed by ACK buffer (KDmedical, Columbia, MD, USA). Then, the cells were separated using 50%/70% Percoll (GE lifescience, Marlborough, MA, USA) gradient, and the tumor-infiltrating lymphocytes (TILs) were obtained between 50% and 70% Percoll. Cell suspensions of blood, spleen and bone marrow were also filtered through Nylon cell strainers and lysed by ACK buffer. Cells were suspended for further analysis.

### 2.5. Flow Cytometry

For cell surface staining, cells were incubated with antibodies for 30 min on ice and then washed with 2% FBS in PBS. SYTOX Blue was used for live/dead cell determination. For intracellular staining, cells were first stained on ice for 30 min with surface staining antibodies. Cells were then fixed and permeabilized with a Fixation/Permeabilization Solution Kit (BD biosciences, Franklin Lakes, NJ, USA) and stained with antibodies for 45 min. For antigen-specific CD8^+^ T-cell staining, cells were washed with 5% FBS and incubated with MHC I Dextramer (H-2 L^d^, SPSYVYHQF, 1: 20) at room temperature (RT) for 20 min, followed by incubation with other antibodies on ice for 30 min. For analysis of cell apoptosis, an Annexin V Apoptosis Detection Kit (ThermoFisher, Waltham, MA, USA) was used according to the manufacturer’s instructions. Samples were analyzed by flow cytometry (BD FACS Fortessa, Franklin Lakes, NJ, USA). Data were analyzed with FlowJo v10.0 software. Antibodies are listed in Supplementary Table S1.

### 2.6. Western Blotting

Proteins were extracted using RIPA buffer mixed with protease inhibitors and phosphatase inhibitors (1:100, GenDEPOT). Protein samples were separated by 10% SDS-PAGE and transferred onto nitrocellulose-blotting membranes. The membranes were blocked with 5% nonfat milk and incubated with primary antibodies overnight followed by incubating with secondary antibody. The reaction was detected by using an enhanced chemiluminescent substrate (ThermoFisher) and signals were captured by X-ray films via Medical Film Processor. Antibodies against β-actin (#4970S) and PARP (#9542S) were obtained from Cell Signaling Technology (Danvers, MA, USA).

### 2.7. RNA Extraction and Real-Time Quantitative PCR

Total RNA was extracted by PureXtract RNAsol RNA Isolation Solution (GenDEPOT) according to the manufacturer’s instructions. RNA was converted to cDNA using a cDNA Reverse Transcription kit (ThermoFisher). Quantitative RT-PCR was performed using SYBR™ Green Master Mix (ThermoFisher) via the StepOnePlus™ Real-Time PCR System. Sequences of primers are shown in Supplementary Table S2. Data were analyzed using the −2^ΔΔct^ method and mRNA expression was normalized to β-actin levels.

### 2.8. MDSCs Isolation

PMN-MDSCs and M-MDSCs were isolated from mice tumors or bone marrow using a Myeloid-Derived Suppressor Cell Isolation Kit (Miltenyi Biotec, Bergisch Gladbach, Germany) according to the manufacturer’s instructions. The purity of PMN-MDSCs or M-MDSCs was greater than 90% (Supplementary Figure S6).

### 2.9. Enzyme-Linked Immunosorbent Assay (ELISA)

The isolated cells were cultured in RPMI 1640 with 10% FBS, 0.1% 2-Mercaptoethanol for 24 h. IL-6, IL-10 and CCL5 concentrations in cell supernatant were examined via ELISA according to the manufacturer’s instructions. IL-6 and IL-10 ELISA kits were obtained from ThermoFisher. The CCL5 ELISA kit was purchased from R&D Systems.

### 2.10. In Vitro T Cells Suppression

T cells were isolated from the spleens of mice using the Mouse T Cell Isolation Kit (Stemcell, Vancouver, BC, Canada) following the manufacturer’s instructions. The isolated T cells were labeled with carboxyfluorescein succinimidyl ester (CFSE, ThermoFisher) at 2 μM. PMN-MDSCs isolated from bone marrow of CT26 tumor-bearing mice were pre-treated with or without oxaliplatin for 20 h. CFSE-labeled T cells were co-cultured with PMN-MDSCs at a 1: 2 ratio for 72 h. Phorbol 12-myristate 13-acetate (PMA, Sigma, St. Louis, MO, USA) at 10 ng/mL and 500 ng/mL ionomycin (Calbiochem, Burlington, MA, USA) were added to stimulate T cells. Flow cytometry was performed to analyze T-cell proliferation.

### 2.11. ELISpot Assay

Multiscreen filter plates (Millipore, Burlington, MA, USA) were pre-coated with interferon (IFN)-γ monoclonal antibody (Thermo Fisher) overnight and blocked with complete medium. Then, 1 x 10^4^ TILs or lymphocytes from popliteal lymph nodes were seeded into the plates and stimulated with GP70 peptide (10 μg/mL) for 36 h. Subsequently, biotin-conjugated IFN-γ antibody (ThermoFisher) was added, followed by incubating with avidin-HRP (ThermoFisher). Spots were visualized by incubation with AEC Substrate solution (BD Biosciences). Plates were scanned using CTL ImmunoSpot Analyzer 6.0.0.2.

### 2.12. Time of Flight Mass Cytometry (CyTOF)

Cell suspension from tumors was stained with metal-tag viability dye and washed with Cell Staining Buffer (Standard BioTools, South San Francisco, CA, USA), followed by staining of surface markers and intracellular markers separately. Cells were then stained with Cell-ID Intercalator Ir (Standard BioTools) at 4 °C overnight. Subsequently, cells were washed and prepared for acquisition with Helios (Standard BioTools). Live-Death was stained with Rh103, allowing Pt195 for detection of Oxaliplatin across a variety cell (sub-)population. Data were analyzed by Cytobank, with preprocessing using PeacoQC to remove low-quality events [36]. Briefly, data were normalized, beads and dead cells were excluded by gating, and the singlet CD45^+^ population was selected to perform t-distributed stochastic neighbor embedding (tSNE) analysis. Subsequently, the downstream analysis gates out diverse immune cell populations based on a guided gating strategy.

### 2.13. Statistical Analysis

Statistical analysis was performed using GraphPad Prism 9.5.1. The normality of distribution and homogeneity of variances were confirmed using the Shapiro–Wilk test and Brown–Forsythe/Bartlett’s tests, respectively. Differences between two groups were analyzed using the two-tailed Student’s *t*-test, and differences among multiple groups were evaluated using one-way ANOVA followed by Tukey’s multiple comparisons test. Animal survival was analyzed via the Log-rank (Mantel–Cox) test. All data are presented as the mean ± standard error. ns indicates not significant, and the difference was considered significant when *p* < 0.05. * *p* < 0.05, ** *p* < 0.01, *** *p* < 0.001.

## 3. Results

### 3.1. Large CT26 Tumors Developed Resistance to µGCGP70 in Murine Models

**Figure 1:**
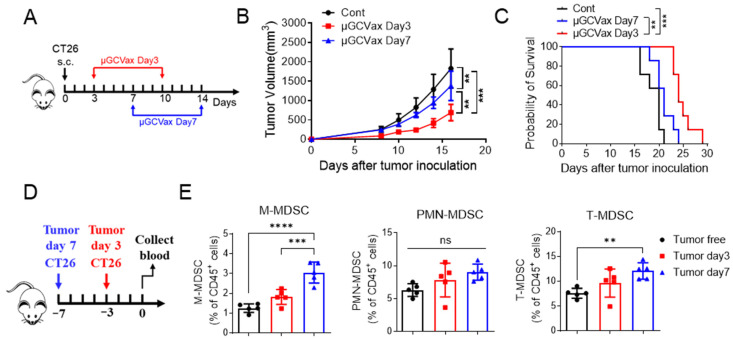
Tumor stage-dependent therapeutic efficacy of µGCGP70 on CT26 tumor growth and MDSC proportions. (**A**) Timeline of treatments in CT26 tumor model. µGCGP70 vaccinations were given on days 3/10 (Red arrows, treatments at early stage) or 7/14 (Blue arrows, treatments at later stage) after subcutaneous CT26 inoculation. (**B**,**C**) Tumor growth and animal survival after treatments (*n* = 7). Cont, control. (**D**) Study timeline. Blood samples were drawn at 7 days or 3 days post CT26 inoculations. Blood samples from tumor-free mice were also collected as control. (**E**) Quantification of M-MDSCs, PMN-MDSCs and T-MDSCs in the CD45^+^ cell population in blood (*n* = 5). One-way ANOVA followed by Tukey’s multiple comparisons test for panels B and E. Survival was analyzed by Log-rank (Mantel–Cox) test for panel (**C**). ns, not significant. ** *p* < 0.01; *** *p* < 0.001, **** *p* < 0.0001.

Although µGCVax has demonstrated robust therapeutic efficacy across multiple murine tumor models [15], its activity has been primarily characterized in early-stage settings. In advanced disease, increasing tumor burden is associated with enhanced MDSC immunosuppression, which can blunt vaccine-induced cytotoxicity. Therefore, we investigated the dependence of µGCGP70 efficacy on tumor size to determine whether, and at what tumor burden, therapeutic benefit is retained. To address this, we evaluated the antitumor effects of the µGCGP70 in CT26 tumors with different sizes. After CT26 inoculation, µGCGP70 was given either on day 3 (average tumor volume ~80 mm^3^) or on day 7 (average tumor volume ~200 mm^3^) (Figure 1A). Administration of µGCGP70 on day 3 resulted in significant tumor regression and markedly improved survival compared with both the day 7-treatment group and vehicle controls (Figure 1B,C). In contrast, initiating µGCGP70 treatment on day 7 produced weaker antitumor effects relative to controls. To investigate potential mechanisms underlying the reduced efficacy in larger tumors, we quantified MDSCs in peripheral blood on days 3 and 7 after tumor inoculation (Figure 1D). We quantified PMN-MDSCs (CD45^+^CD11b^+^Ly6G^+^) and M-MDSCs (CD45^+^CD11b^+^Ly6C^high^) as a proportion of total CD45^+^ leukocytes. The representative gating strategy is shown in Supplementary Figure S1A. Total MDSCs (T-MDSCs) were more abundant in day 7 tumor-bearing mice than in control groups. Within this population, M-MDSCs were significantly elevated compared with tumor-free mice and mice at day 3 post-inoculation (Figure 1E; Supplementary Figure S1B), whereas PMN-MDSCs only showed a slight numerical increase on day 7. However, the frequencies of circulating T cells, including CD4^+^ and CD8^+^ subsets, remained largely unchanged (Supplementary Figure S1C,D). These results indicate that µGCGP70 efficacy is tumor-burden dependent and is attenuated in larger, established tumors that exhibit a marked expansion of MDSCs, especially M-MDSCs, consistent with heightened myeloid-driven immunosuppression.

### 3.2. Oxaliplatin Combined with µGCGP70 Synergistically Inhibited CT26 Tumor Growth

**Figure 2:**
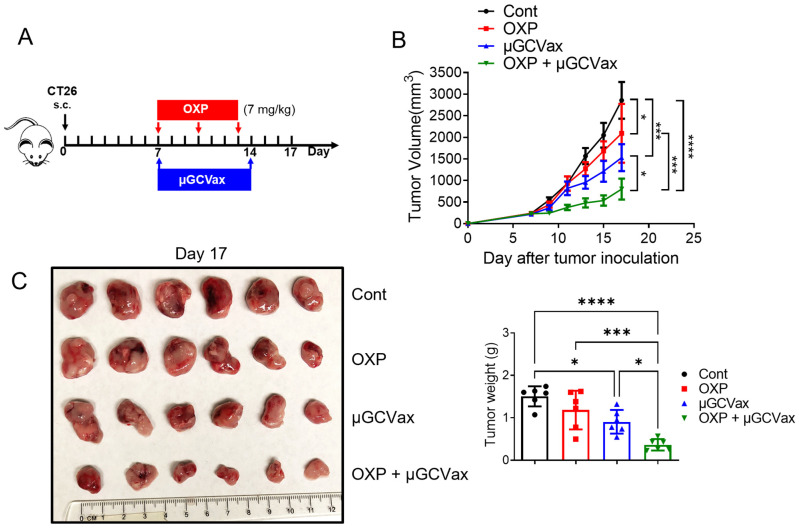
Oxaliplatin combined with µGCGP70 synergistically inhibited CT26 tumor growth. (**A**) Treatments scheme. Seven days after subcutaneous tumor inoculation (Tumor volume: ~200 mm^3^), mice received oxaliplatin via i.v. injection and µGCGP70 via foodpad injection. Oxaliplatin was injected every 3 days 3 times and µGCGP70 was injected twice every 7 days (*n* = 6). (**B**) Tumor growth curve after treatment. (**C**) Mice were euthanized on day 17, and tumor tissues were collected and weighed. Cont, control; OXP, oxaliplatin; One-way ANOVA followed by Tukey’s multiple comparisons test for panels (**B**,**C**). * *p* < 0.05, *** *p* < 0.001, **** *p* < 0.0001.

Based on these observations, we hypothesized that relieving MDSC-mediated immunosuppression would restore µGCGP70 efficacy in established tumors. Since oxaliplatin has been reported to deplete MDSCs and alleviate the immunosuppressive TME [29,30,31,32], we next tested whether oxaliplatin could enhance the antitumor efficacy of µGCGP70. We evaluated a combination therapy in mice bearing established, large CT26 tumors. When tumor volumes reached approximately 200 mm^3^ at day 7, mice were treated with a low dosage of oxaliplatin (7 mg/kg), µGCGP70 alone, or their combination (Figure 2A). Neither oxaliplatin nor µGCGP70 monotherapy produced robust antitumor effects. In contrast, combined treatment with oxaliplatin and µGCGP70 resulted in a pronounced inhibition of tumor growth and significantly extended overall survival compared with both the control and monotherapy groups (Figure 2B,C). Analysis of relative body weight changes indicated that mice tolerated both monotherapies and the combination regimen well, with body weight loss remaining below 10% and no obvious behavioral changes observed during treatment (Supplementary Figure S2A). Collectively, these results demonstrate that oxaliplatin synergizes with the µGCGP70 to effectively suppress established CT26 tumor growth.

### 3.3. Oxaliplatin Increased T-Cell Populations and Promoted Antigen Specific CD8^+^ T-Cell Infiltration into Tumor

**Figure 3:**
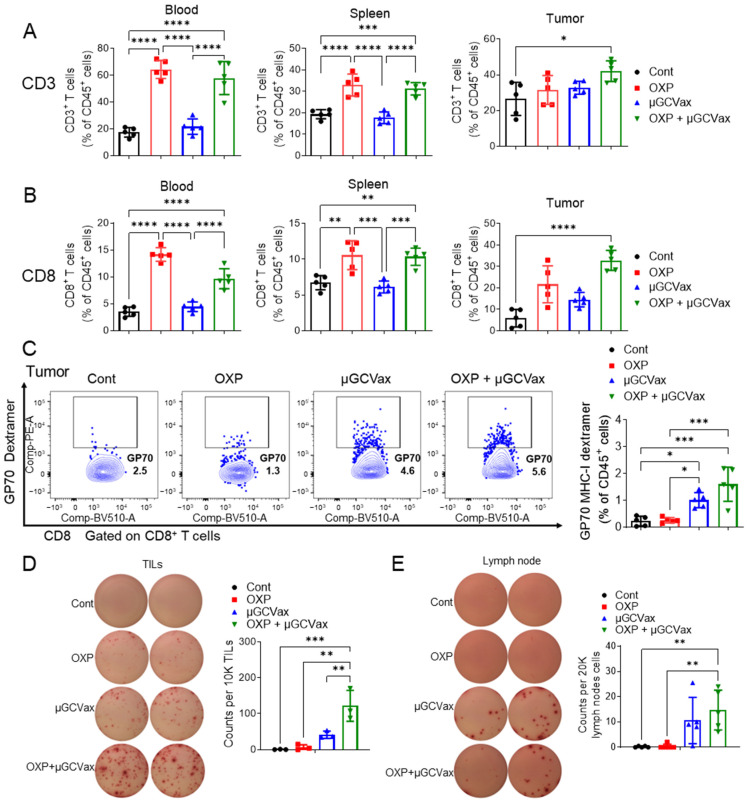
Oxaliplatin increased T-cell population and promoted tumor infiltration of antigen-specific CD8^+^ T cells. CT26-bearing BALB/c mice received treatments on day 7 post-inoculation and were euthanized at day 17 to collect blood, spleen and tumor tissues and prepare single cell suspensions. The frequency of total CD3^+^ T cells (**A**) and CD8^+^ subset in CD45^+^ leukocytes (**B**) in blood, spleen and tumor were evaluated by flow cytometry. (**C**) The proportion of GP70-specific CD8^+^ T cells in CD45^+^ cells in tumors was evaluated by flow cytometry. (**D**) TILs were pulsed ex vivo with GP70 peptide (10 μg/mL) for 36 h, and IFN-γ–producing cells were quantified by ELISpot analysis (*n* = 5 mice per group). Cont, control; OXP, oxaliplatin; TILs, tumor infiltrating lymphocytes. One-way ANOVA followed by Tukey’s multiple comparisons test for panels (**A**–**E**). * *p* < 0.05, ** *p* < 0.01, *** *p* < 0.001, **** *p* < 0.0001.

Having observed strong tumor control and improved survival with the combination regimen, we next asked whether oxaliplatin enhances µGCGP70 efficacy by augmenting intratumoral T-cell infiltration and antigen-specific CD8^+^ T-cell function. To address this, we evaluated T-cell responses in blood, spleen, and tumors 10 days after the initial treatment. We quantified CD3 (CD45^+^CD3^+^), CD8 (CD45^+^CD3^+^CD8^+^) and GP70-specific CD8^+^ T (CD45^+^CD3^+^CD8^+^GP70^+^) cells as a proportion of total CD45^+^ leukocytes. The representative gating strategy and fluorescence minus one (FMO) control for the GP70 dextramer are shown in Supplementary Figure S3A. The total CD3^+^ T cell and the CD8^+^ subset within CD45^+^ leukocytes increased in blood and spleen in both the oxaliplatin monotherapy and combination therapy groups. However, in tumor tissues, significant increases in total CD3^+^ and CD8^+^ T cells were observed only in the combination therapy group (Figure 3A,B; Supplementary Figure S3B,C). Considering that MDSCs can restrict T-cell infiltration and function, we further examined the proportion of GP70 antigen-specific CD8^+^ T cells in tumors. Treatment with µGCGP70 alone or in combination with oxaliplatin increased the frequency of GP70-specific CD8^+^ T cells among CD45^+^ cells, with the combination therapy inducing the highest frequency of GP70-specific CD8^+^ T cells (Figure 3C). Consistent with these infiltration data, functional GP70-specific CD8^+^ responses were increased as quantified via the IFN-γ ELISpot assay following GP70 stimulation. The frequency of IFN-γ-producing cells in tumors was significantly elevated in both the µGCGP70 alone and combination groups (Figure 3D). Notably, the combination therapy group exhibited a further significant increase compared with µGCGP70 alone, indicating that oxaliplatin enhanced the antitumor activity of CD8^+^ T cells. Since µGCGP70 was administered via footpad injection, we analyzed immune responses in the draining popliteal lymph nodes to determine whether oxaliplatin influenced vaccine-induced immunity. The percentages of IFN-γ^+^ cells in the popliteal lymph nodes were similarly and robustly increased in both the µGCGP70 alone and combination groups (Figure 3E), indicating that oxaliplatin did not alter the initial immune response induced by the µGCGP70. Collectively, these results demonstrate that oxaliplatin increases circulating T cells in CT26 tumor-bearing mice and promotes the infiltration and antitumor function of antigen-specific CD8^+^ T cells within tumors, thereby enhancing the overall efficacy of µGCGP70 therapy.

### 3.4. Oxaliplatin Depleted MDSCs in CT26 Tumor-Bearing Mice

**Figure 4:**
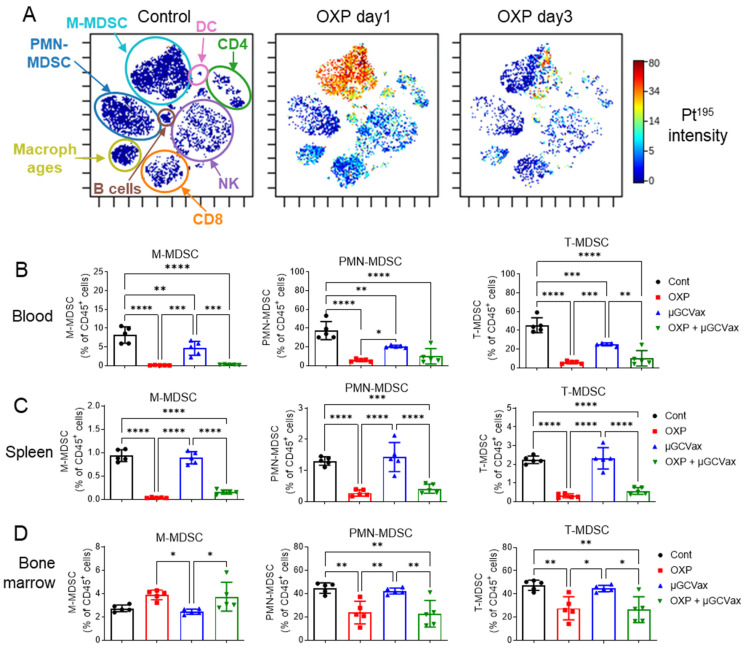
Oxaliplatin depleted MDSCs in CT26 tumor-bearing mice. (**A**) CyTOF analysis of intratumoral leukocytes at 24 h or 72 h after oxaliplatin (7 mg/kg) treatment. Color shows Platinum (Pt) signal intensity in cells (blue: low Pt intensity; red: high Pt intensity). M-MDSCs, CD45^+^CD11b^+^Ly6C^hi^; PMN-MDSCs, CD45^+^CD11b^+^Ly6G^+^; macrophages, CD45^+^CD11b^+^ F4/80^+^; NK cells, natural killer cell, CD45^+^CD3^-^Nkp46^+^. B cells, B220^+^; DCs: CD45^+^F4/80^-^MHCII^+^CD11c^+^. (**B**–**D**) The proportions of MDSCs in blood, spleen and bone marrow were evaluated by flow cytometry 17 days after tumor inoculation. M-MDSCs, identified as CD45^+^CD11b^+^Ly6C^+^; PMN-MDSCs, identified as CD45^+^CD11b^+^Ly6G^+^; T-MDSCs (Total MDSCs), M-MDSCs plus PMN-MDSCs. *n* = 5 mice per group. Cont, control; OXP, oxaliplatin; One-way ANOVA followed by Tukey’s multiple comparisons test for panels (**B**–**D**). ns, not significant. * *p* < 0.05, ** *p* < 0.01, *** *p* < 0.001, **** *p* < 0.0001.

Given that oxaliplatin potentiated µGCGP70 and elicited potent antigen-specific antitumor T-cell immunity within tumors rather than in lymphatic tissues, we next investigated in detail how oxaliplatin reshapes the TME, focusing on its effects on different cell populations and the following phenotypic changes after treatment in the tumor. To this end, we employed CyTOF to quantify platinum (^195^Pt) signals as a surrogate marker of oxaliplatin uptake in different populations of intratumoral immune cells isolated from control and oxaliplatin-treated mice. CT26 tumors were harvested 1 or 3 days after intravenous administration of oxaliplatin. Pt-positive cells were observed in the oxaliplatin treatment groups, with the strongest signal detected in M-MDSCs (CD11b^+^Ly6C^hi^) at 1 day after oxaliplatin single administration, indicating selective uptake of oxaliplatin by M-MDSCs among intratumoral immune cells in vivo (Figure 4A). Notably, the ^195^Pt signal markedly decreased by day 3 post-administration (Figure 4A), suggesting rapid clearance or turnover of oxaliplatin within the TME. This temporal pattern is consistent with the dosing regimen used in the efficacy studies, in which oxaliplatin was administered every 3 days to maintain sufficient intratumoral drug exposure (Figure 2A).

Given the preferential uptake of oxaliplatin by M-MDSCs, we then analyzed treatment-induced phenotypic changes of MDSCs by flow cytometry. MDSCs in the blood, spleen, and bone marrow were quantified at 4 days after the third dose of oxaliplatin. To minimize confounding effects of tumor burden on immune cell composition, size-matched tumors were compared across treatment groups [35]. We quantified PMN-MDSCs (CD45^+^CD11b^+^Ly6G^+^) and M-MDSCs (CD45^+^CD11b^+^Ly6C^high^) as a proportion of total CD45^+^ leukocytes. The gating strategy is shown in Supplementary Figure S4A and representative flow cytometry plots in Supplementary Figure S4B are gated on CD45^+^CD11b^+^ cells. Oxaliplatin markedly reduced the proportion of M-MDSCs (CD45^+^CD11b^+^Ly6C^high^) in the blood and spleen in both the oxaliplatin monotherapy group and the combination therapy group (Figure 4B,C; Supplementary Figure S4B,C). Oxaliplatin also significantly decreased the proportion of PMN-MDSCs (CD45^+^CD11b^+^Ly6G^+^) in the blood and spleen in both treatment groups. Analysis of T-MDSCs revealed a similar trend, with oxaliplatin treatment leading to reduced T-MDSC proportions in the blood and spleen in both the monotherapy and combination groups.

Since MDSCs originate from the bone marrow [21,37], we next assessed MDSC populations in this compartment. Oxaliplatin treatment significantly reduced the proportions of PMN-MDSCs and T-MDSCs among CD45^+^ cells in the bone marrow in both the oxaliplatin alone and combination therapy groups (Figure 4D; Supplementary Figure S4D). Interestingly, the proportion of M-MDSCs in the bone marrow was increased following oxaliplatin treatment in both groups. This likely reflects compensatory emergency myelopoiesis in response to oxaliplatin-induced depletion of peripheral MDSCs. Taken together, the peripheral depletion of M-MDSCs, concurrent with their accumulation in the bone marrow, suggests that oxaliplatin modulates M-MDSC differentiation and function rather than simply causing systemic elimination.

### 3.5. Oxaliplatin Regulated M-MDSCs Function and Differentiation in Tumor

**Figure 5:**
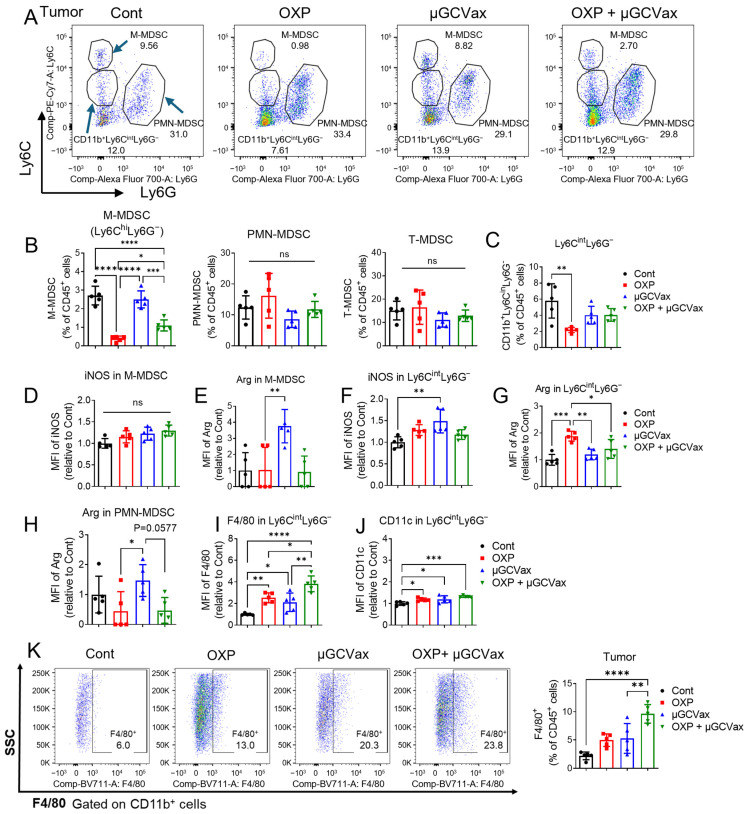
Oxaliplatin regulated M-MDSCs function and differentiation in tumor. (**A**,**B**) The proportions of MDSCs in tumors were evaluated by flow cytometry 17 days after tumor inoculation. M-MDSCs, identified as CD45^+^CD11b^+^Ly6C^high^Ly6G^−^; PMN-MDSCs, identified as CD45^+^CD11b^+^Ly6G^+^; T-MDSCs (Total MDSCs), M-MDSCs plus PMN-MDSCs. *n* = 5 mice per group. (**C**) The proportions of CD45^+^CD11b^+^Ly6C^int^Ly6G^-^ in tumors. (**D**,**E**) Median fluorescence intensity (MFI) of iNOS (**D**) and Arg1 (**E**) in the M-MDSCs population. (**F**,**G**) MFI of iNOS (**F**) and Arg1 (**G**) in the CD45^+^CD11b^+^Ly6C^int^Ly6G^-^ population. (**H**) MFI of Arg1 in the PMN-MDSC population. (**I**,**J**) MFI of F4/80 (**I**) and CD11c (**J**) in the CD45^+^CD11b^+^Ly6C^int^Ly6G^-^ population. (**K**) The proportions of F4/80^+^ cells in CD45^+^CD11b^+^ cells in tumors were evaluated by flow cytometry. One-way ANOVA followed by Tukey’s multiple comparisons test for panels (**B**–**K**). ns, not significant. * *p* < 0.05, ** *p* < 0.01, *** *p* < 0.001, **** *p* < 0.0001.

We further analyzed MDSC populations within tumors. Oxaliplatin significantly reduced the proportion of M-MDSCs (CD11b^+^Ly6C^high^Ly6G^-^) in both the oxaliplatin alone and combination treatment groups (Figure 5A,B), consistent with changes observed in the blood and spleen (Figure 4B,C). In contrast, the frequencies of PMN-MDSCs and total MDSCs (T-MDSCs) within tumors were not significantly affected (Figure 5A,B). We further identified a population of tumor-infiltrating myeloid cells with intermediate Ly6C expression (CD11b^+^Ly6C^int^Ly6G^−^). This Ly6C^int^ population arises from the differentiation or transitional state of M-MDSCs (CD11b^+^Ly6C^hi^Ly6G^−^) as they migrate into the TME and begin to form the tissue-resident phenotype, progressing toward macrophage- or dendritic cell (DC)-like differentiation [38]. Interestingly, while oxaliplatin significantly reduced the frequency of CD11b^+^Ly6C^int^Ly6G^−^ cells compared with the control group, neither the µGCGP70 alone nor the combination treatment produced a similar decrease (Figure 5C).

To investigate the functional state of M-MDSCs, we assessed the expression of inducible nitric oxide synthase (iNOS) and arginase 1 (Arg1), key markers associated with immunosuppressive activity in M-MDSCs [39]. Within the M-MDSC population, iNOS expression levels were similar across all groups (Figure 5D). Interestingly, the nanovaccine alone significantly increased Arg1 expression, whereas the combination with oxaliplatin did not (Figure 5E), suggesting that while the µGCGP70 can enhance Arg1 expression, oxaliplatin may counteract this effect. Within the CD11b^+^Ly6C^int^Ly6G^−^ population, the µGCGP70 significantly enhanced iNOS expression compared with the control group (Figure 5F), whereas the combination with oxaliplatin did not. On the other hand, oxaliplatin alone increased Arg1 expression in this population, but this effect was absent in the combination group (Figure 5G). Additionally, the µGCGP70 also elevated Arg1 expression in the PMN-MDSC population; however, this effect was significantly reduced in the combination group (Figure 5H). Collectively, these results indicate that the µGCGP70 and oxaliplatin differentially modulate the functional state of MDSC subsets, and that their combination can counterbalance the individual effects of each treatment, resulting in a less immunosuppressive TME.

Given that CD11b^+^Ly6C^int^Ly6G^−^ cells can differentiate into macrophages or DCs, we next examined the expression of F4/80 and CD11c within this population. Both oxaliplatin and the µGCGP70 significantly increased F4/80 expression (approximately 2-fold) compared with the control group in CD11b^+^Ly6C^int^Ly6G^−^ cells, with the combination treatment producing the greatest increase (approximately 4-fold) (Figure 5I). CD11c expression was also elevated; however, the magnitude of this increase was more modest, reaching approximately 1.3-fold in the combination group relative to controls (Figure 5J). These results indicate that the combination treatment preferentially promotes macrophage differentiation of CD11b^+^Ly6C^int^Ly6G^−^ cells within the TME, with a lesser effect on DCs differentiation.

We next analyzed the proportion of F4/80^+^ macrophages within the CD45^+^ cell population and found that it was significantly increased in the combination therapy group in both the bone marrow and tumors (Figure 5K, Supplementary Figure S5A), whereas no significant change was observed in the oxaliplatin monotherapy group. Further phenotypic analysis revealed that the combination of oxaliplatin and µGCGP70 significantly increased M1 macrophages in the bone marrow, without a corresponding increase in M2 macrophages (Supplementary Figure S5B). Although a modest increase in M1 macrophages was also observed in the tumor following combination treatment, this change did not reach statistical significance (Supplementary Figure S5C). Interestingly, M2 macrophages were significantly increased within tumors following treatment with either oxaliplatin alone or the combination regimen, which may contribute to therapeutic resistance to the combination therapy (Supplementary Figure S5C). Collectively, these results demonstrate that oxaliplatin modulates M-MDSC differentiation and macrophage polarization, and that its combination with the µGCGP70 reshapes the myeloid compartment toward a less immunosuppressive, though not fully reprogrammed, TME.

### 3.6. Oxaliplatin Selectively Depleted M-MDSCs In Vitro

**Figure 6:**
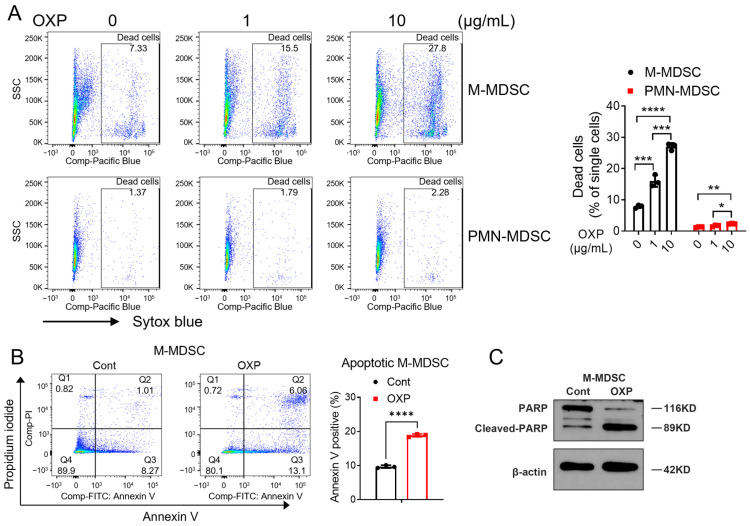
Oxaliplatin selectively depleted M-MDSCs ex vivo. (**A**) M-MDSCs and PMN-MDSCs were isolated from the bone marrow of CT26 tumor-bearing mice and treated with oxaliplatin for 20 h. Cell viability was assessed by SYTOX Blue staining followed by flow cytometric analysis. (**B**) M-MDSCs were then stained with Annexin V and propidium iodide, and apoptosis was analyzed by flow cytometry. (**C**) Expression of PARP, cleaved PARP, and β-actin was analyzed by Western blot (**C**). Cont, control; OXP, oxaliplatin. One-way ANOVA followed by Tukey’s multiple comparisons test for panel (**A**); two-tailed Student’s *t* Test for panel (**B**). ns, not significant. * *p* < 0.05, ** *p* < 0.01, *** *p* < 0.001, **** *p* < 0.0001.

To further confirm the selective effect of oxaliplatin on M-MDSCs, M-MDSCs and PMN-MDSCs were isolated from bone marrow and treated with oxaliplatin in vitro. Isolated M-MDSCs and PMN-MDSCs showed purities of >90% and >99%, respectively, confirming the successful isolation of these populations (Supplementary Figure S6A,B). M-MDSCs are more vulnerable to oxaliplatin at 1 or 10 μg/mL, while after 24 h treatment, while PMN-MDSCs are less affected (Figure 6A). Next, we measured oxaliplatin-induced apoptosis in M-MDSCs following treatment. Annexin V staining revealed a significant increase in apoptotic M-MDSCs after oxaliplatin exposure (Figure 6B). Consistently, Western blot analysis showed markedly increased cleaved-PARP levels in M-MDSCs, a well-established marker of apoptosis (Figure 6C). Together, these findings demonstrate that oxaliplatin selectively depletes M-MDSCs both in vivo and in vitro by inducing apoptosis.

### 3.7. Oxaliplatin Impaired T-Cell Suppression Function of PMN-MDSCs In Vivo and In Vitro

Although oxaliplatin did not affect the viability of PMN-MDSCs in vitro, we investigated whether it could modulate their immunosuppressive function. Isolated PMN-MDSCs were pretreated with oxaliplatin (5 μg/mL) for 24 h, a concentration that does not affect cell viability (Figure 6A), then washed to remove the drug and co-cultured with T cells. As expected, untreated PMN-MDSCs strongly suppressed the proliferation of both CD3^+^ and CD8^+^ T cells (Supplementary Figure S7A). In contrast, PMN-MDSCs pretreated with oxaliplatin partially lost their suppressive activity, with a more pronounced effect on CD8^+^ T cells (Supplementary Figure S7A). Consistently, proliferating T cells formed prominent clusters when cultured alone with stimulation, whereas co-culture with untreated PMN-MDSCs abolished cluster formation (Supplementary Figure S7B). Notably, PMN-MDSCs pretreated with oxaliplatin partially restored T-cell clustering. To assess this effect in vivo, CT26 tumor-bearing mice were treated with oxaliplatin (Supplementary Figure S7C), and PMN-MDSCs were isolated from tumors and cultured for 24 h. mRNA levels of immunosuppressive markers, including CCL2, CCL5, Arg1, and NOX2, were significantly reduced in PMN-MDSCs from oxaliplatin-treated mice compared with controls (Supplementary Figure S7D). Reduced CCL5 expression was further confirmed by ELISA (Supplementary Figure S7E). Additionally, PMN-MDSCs from the oxaliplatin group expressed lower levels of IL-6 and IL-10, further indicating impaired immunosuppressive function (Supplementary Figure S7E). Oxaliplatin mostly accumulated in M-MDSCs rather than PMN-MDSCs (Figure 4A). Therefore, we hypothesize that the attenuated immunosuppresive activity of PMN-MDSCs is indirectly mediated through oxaliplatin-induced reprograming of M-MDSCs and tumor-associated macrophages (TAMs). Intratumoral myeloid-derived factors such as CXCL1, IL-10, and TGF-β have been implicated in regulating PMN-MDSC immunosppressive function [40,41]. The mechanistic interplay among oxaliplatin, PMN-MDSCs, and M-MDSCs remains to be defined. Together, these results demonstrate that oxaliplatin can attenuate the T-cell-suppressive function of PMN-MDSCs both in vitro and in vivo, without affecting their viability.

## 4. Discussion

In our previous study, we developed a µGCVax vaccine platform capable of inducing potent antigen-specific cytotoxic CD8^+^ T cells and stimulating antitumor immunity across multiple cancer models [15]. In the current study, we observed that the antitumor efficacy of this µGCVax was reduced in larger CT26 tumors (Figure 1B,C), a scenario that parallels clinical challenges where most patients present with advanced-stage tumors and immunotherapy often shows limited efficacy. This highlights the need to combine with other modalities to overcome tumor-induced resistance.

Therapeutic cancer vaccines have gained momentum following the recent encouraging results from the mRNA-4157/V940 vaccine in the phase 2b KEYNOTE-942 trial [42]. However, although their applications have been evaluated in clinical trials against CRCs and can elicit measurable antigen-specific T-cell responses, clinical benefits are mostly often observed in patients with lower baseline tumor burden, including those treated in adjuvant therapies [9,14], which is a common problem therapeutic vaccines face, including our platform (Figure 1A–C). It indicates the need for developing strategies to overcome the immunosuppressive TME characteristic of MSS CRCs [43]. MSS CRCs are characterized by less immunogenicity, lower neoantigen burden, and immunosuppressive TMEs enriched in suppressive cell populations, including regulatory T cells, tumor-associated macrophages, and MDSCs, which collectively restrain effective vaccine-induced T-cell immunity. Therefore, strategies that alleviate TME-mediated immunosuppression are essential to enhance the therapeutic efficacy of cancer vaccines in advanced CRC.

Oxaliplatin is widely used in CRC and has been reported to inhibit MDSCs, thereby alleviating immunosuppressive TMEs [31,33,44,45]. However, its effects on circulating and intratumoral MDSC subsets remain poorly characterized. In our current study, we showed that oxaliplatin can decrease the number of MDSCs, especially M-MDSCs, in CT26 tumor-bearing mice. We observed that the circulating M-MDSCs increased with tumor progression, with PMN-MDSCs showing a similar trend (Figure 1E,F), consistent with their reported potent immunosuppressive activity at tumor sites, often exceeding that of PMN-MDSCs [46,47,48]. Interestingly, oxaliplatin is mostly taken up and affects M-MDSCs among leukocytes in TMEs (Figure 4A). It has been reported that oxaliplatin tends to accumulate in tumor stromal and necrotic regions, where myeloid cells are often enriched [49,50], but its preferential accumulation in M-MDSCs in CRC TMEs was less reported. The selective effects of oxaliplatin on M-MDSCs may represent a key mechanism by which this agent alleviates immunosuppression and reshapes the TME. Consequently, we also evaluated its impact on M-MDSC viability and function, and determined whether it preferentially targets M-MDSCs in vivo and in vitro.

Beyond numerical depletion, oxaliplatin exerted profound effects on MDSC function and differentiation within the TME. Oxaliplatin not only reduces the abundance of M-MDSCs but also reshapes their functional phenotype, modulating the balance between immunosuppressive and immunostimulatory programs, and promoted the maturation and differentiation of immature M-MDSCs (Figure 5B–K). For example, while the µGCVax alone paradoxically upregulates immunosuppressive markers such as Arg1 and iNOS, the addition of oxaliplatin effectively blunts this feedback, creating a significantly less hostile TME (Figure 5E,F). This “counterbalancing effect” is critical, as it ensures that the pro-inflammatory signals induced by the vaccine are not immediately neutralized by a concomitant rise in myeloid-mediated suppression. Oxaliplatin causes intratumoral accumulation of total macrophages, including both M1 and M2 populations (Figure 5K, Supplementary Figure S5C), potentially contributing to therapeutic resistance. These findings underscore the context-dependent nature of myeloid reprogramming, where systemic changes do not always translate into fully immunostimulatory TMEs. Therefore, combining oxaliplatin with strategies that selectively target or repolarize M2 macrophages could further enhance antitumor efficacy. Additional strategies to inhibit M2 macrophages, such as reagents targeting CSF1R and PI3Kγ, will be considered in future treatment regimens [51,52]. Additionally, the mechanisms underlying the altered iNOS and Arg1 expression in MDSCs following oxaliplatin treatment remain unclear and warrant further investigation, as they may reveal new opportunities to fine-tune myeloid responses in chemo-immunotherapy combinations.

In our study, oxaliplatin reduced overall MDSCs, particularly PMN-MDSCs in bone marrow and circulation, whereas intratumoral PMN-MDSCs were mostly oxaliplatin-free and were not decreased in abundance, likely reflecting their lower phagocytosis and micropinocytosis capability (Figure 4A). Notably, neutrophils recruited by combinational regimens of oxaliplatin and immunotherapeutics can adopt an N1-like, pro-immune phenotype [53]. Indeed, intratumoral PMN-MDSCs in the combination group exhibited a lower Arg1 expression level (Figure 5H). In vitro studies confirmed that PMN-MDSCs were less vulnerable to direct oxaliplatin killing, but their T-cell suppression function was diminished, coming with downregulation of N2 pro-tumor markers such as Arg1, NOX2, CCL2, CCL5, IL-6 and IL-10 (Supplementary Figure S7C,D). Further studies are needed to dissect the precise mechanisms underlying these indirect effects on PMN-MDSC function.

Reprogramming immunosuppressive myeloid cells by oxaliplatin relieves T-cell inhibition and promotes T-cell recruitment and activation within TMEs, thereby enhancing antitumor efficacy. We observed increased T-cell populations in oxaliplatin-treated CT26 tumor-bearing mice (Figure 3A,B). We further investigated the synergistic effect when combining oxaliplatin with µGCVax and found that there were more antigen-specific CD8^+^ T cells present in the TME after combination therapy. Additionally, secretion of IFN-γ, which is an activation marker of cytotoxic T cells, was significantly higher in the combination group. This improved immune response was consistent with the inhibition of tumor growth in the combination group (Figure 2B,C). We speculate that the synergistic effect between oxaliplatin and µGCVax arises from oxaliplatin-mediated reduction of MDSCs and their immunosuppressive function, thereby permitting enhanced tumor infiltration and antitumor activity of antigen-specific CD8^+^ T cells. Oxaliplatin was also reported to induce ICD of cancer cells, stimulate antigen-presenting cells to process tumor-associated antigens and present them to T cells, resulting in stimulation of T cells and an enhanced cytotoxic effect [54]. As a moderate increase in DC population was observed after treatments (Figure 5J), we anticipate that the ICD effects of oxaliplatin contribute to the overall therapeutic outcome, but to a lesser extent when combining with vaccines using defined antigens with high immunogenicity. It needs to be further investigated in future studies on DC activity and antigen-spreading assays.

Although the undifferentiated CT26 model, which is relatively more immunogenic and lacks typical driver gene mutations seen in human CRC, does not fully recapitulate all aspects of human MSS CRC, it remains a widely used and useful syngeneic murine CRC model with pMMR/MSS-like features for mechanistic studies [55]. Human CRC is highly heterogeneous at both intertumoral and intratumoral levels, and many clinical tumors contain mixed Consensus Molecular Subtypes (CMSs) programs within the same lesion, which cannot be fully modeled by a relatively homogeneous cell line-derived system such as CT26 [56,57]. Importantly, however, CT26 is still highly relevant for the current study because it induces a robust immunosuppressive myeloid tumor microenvironment, including granulocytic and monocytic MDSCs that suppress T-cell responses through arginase-1- and iNOS-associated pathways [58,59]. As the major focus of this research is the regulation of suppressive myeloid cells and TAM repolarization, CT26 provides a reasonable and biologically relevant in vivo model for this purpose. Future studies in complementary models with greater clinical relevance, such as transgenic CRC models and humanized Patient-Derived Xenograft (PDX) models, will be valuable to further extend the translational significance of these findings [55].

In summary, combining oxaliplatin with a µGCVax nanovaccine generates synergistic antitumor immunity in large, advanced CT26 tumors. Mechanistically, oxaliplatin selectively depletes M-MDSCs and impairs PMN-MDSC function. This reshapes the TME to reduce immunosuppression and enable robust CD8^+^ T-cell activation and tumor control. These results highlight the potential of chemo-immunotherapy combinations to overcome immunosuppressive TMEs and suggest that this strategy may represent a promising therapeutic option for CRC patients.

## Data Availability

The original contributions presented in the study are included in the article, further inquiries can be directed to the corresponding author.

## Supplementary Materials

The following supporting information can be downloaded at: https://www.mdpi.com/article/10.3390/jfb17040185/s1. Table S1: The antibodies used for flow cytometry. Table S2: The primer sequences for RT-qPCR. Figure S1: The T cell percentage at different tumor stages. Figure S2: The relative body weight change of CT26 tumor-bearing mice after treatments. Figure S3: Combination of oxaliplatin and μGCVax enhances T cell infiltration and increases tumor-specific CD8^+^ T cells. Figure S4: Oxaliplatin depleted MDSCs in CT26 tumor-bearing mice. Figure S5: Oxaliplatin modulated macrophage polarization. Figure S6: Purity of isolated M-MDSCs and PMN-MDSCs. Figure S7: Oxaliplatin suppressed the function of PMN-MDSCs in vivo and in vitro.
