# Supplementary material for: Potentiation of a Porous Silicon Therapeutic Vaccine in Colorectal Cancer via Oxaliplatin-Mediated Regulation of Myeloid-Driven Immunosuppression
Source: J Funct Biomater. 2026 Apr 10;17(4):185. doi: 10.3390/jfb17040185 (PMC13117512; doi:10.3390/jfb17040185)
Supplement: Supplementary file 1 [file jfb-17-00185-s001.zip › jfb-4185834-supplementary.pdf]

SUPPLEMENTARY DATA

Table S1: The antibodies used for flow cytometry.

Antibody	Clone	Manufacture	Cat#	Dilution
APC anti-mouse CD45 Antibody	30-F11	Biolegend	103112	1:400
PerCP-Cyanine5.5 Anti-Mouse CD3e	145-2C11	TonboBiosciences	65-0031-U100	1:200
APC-Cy™7 Rat Anti-Mouse CD45	30-F11	BDbiosciences	557659	1:400
APC-Cyanine7 Anti-Mouse Ly-6G	1A8	TonboBiosciences	25-1276-U100	1:200
PE Anti-Human/Mouse CD11b	M1/70	TonboBiosciences	50-0112-U100	1:400
PE anti-mouse CD4	GK1.5	Biolegend	100408	1:400
Pacific Blue CD45 Rat Anti-Mouse	30-F11	Thermofisher	MCD4528	1:400
BV711 Rat Anti-Mouse F4/80	T45-2342	BDbiosciences	565612	1:400
BV510 Rat Anti-Mouse CD8a	53-6.7	BDbiosciences	563068	1:400
PE/Cyanine7 anti-mouse Ly-6C	HK1.4	Biolegend	128018	1:200
Alexa Fluor® 700 anti-mouse Ly-6G	1A8	Biolegend	127622	1:200
Alexa Fluor® 700 anti-mouse Ly-6C	HK1.4	Biolegend	128024	1:200
FITC anti-mouse/human CD11b	M1/70	Biolegend	101206	1:400
FITC CD4 Monoclonal Antibody	GK1.5	Thermofisher	11-0041-82	1:400
FITC Mouse Anti-iNOS/NOS Type II	6/iNOS/NOS Type II	BDbiosciences	610331	1:200
H-2 L^d (SPSYVYHQF), PE	—	Immudex	JG3294 - PE	1:20
SYTOX™ Blue Dead Cell Stain	—	Thermofisher	S34857	1:1000

Table S2: The primer sequences for RT-qPCR.

Primer	Sequence (5' to 3')
CCL2-F	AGCCAACTCTCACTGAAGCC
CCL2-R	GGACCCATTCCTTCTTGGGG
CCL5-F	TGCTCCAATCTTGCAGTCGT
CCL5-R	GCAAGCAATGACAGGGAAGC
β-actin-F	GGTGTGATGGTGGGAATGGG
β-actin-R	ACGGTTGGCCTTAGGGTTCAG
ARG1-F	AACACGGCAGTGGCTTTAACCT
ARG1-R	GTGATGCCCCAGATGGTTTTC
NOX2-F	GACCCAGATGCAGGAAAGGAA
NOX2-R	TCATGGTGCACAGCAAAGTGAT

Supplementary Figure legends

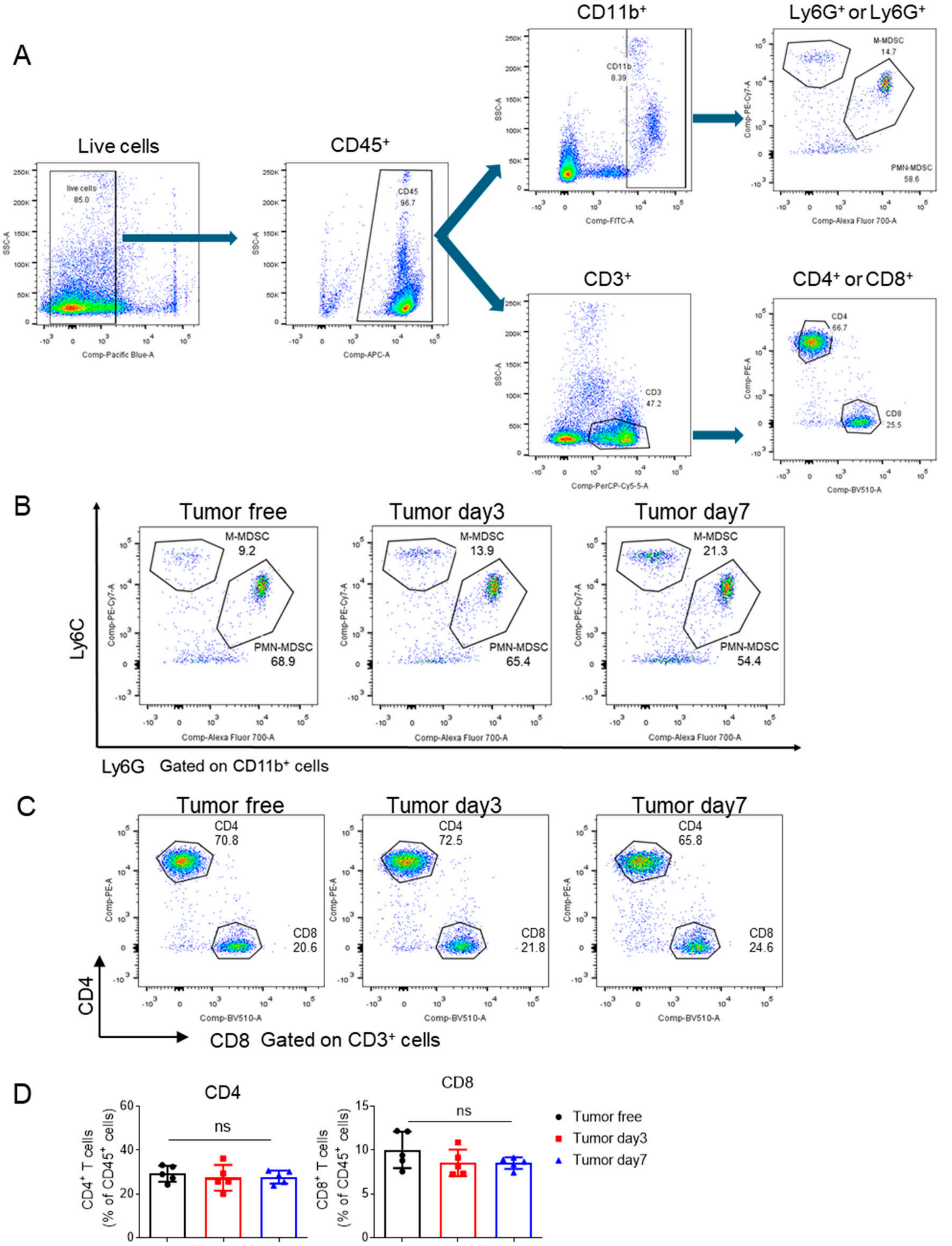

Figure S1. The T cell percentage at different tumor stages. (**A**) Representative gating strategy for the T cells and MDSCs in blood. **(B)** The proportions of M-MDSCs ($CD45^+CD11b^+Ly6C^+$), PMN-MDSCs ($CD45^+CD11b^+Ly6G^+$) and T-MDSCs (Total MDSCs, M-MDSCs plus PMN-MDSCs) were determined by flow cytometry. **(C)** Representative flow cytometry plots of CD4 and CD8 T cells in blood. Cells in the $CD3^+$ gate are shown. **(D)** Proportions of $CD4^+$ and $CD8^+$ T cells in the $CD45^+$ cell population in blood. CD4 T cells, identified as

$CD45^{+}CD3^{+}CD4^{+}$; CD8 T cells, identified as $CD45^{+}CD3^{+}CD8^{+}$; n = 5 mice per group. One-way ANOVA followed by Tukey's multiple comparisons test for panel D. ns, not significant.

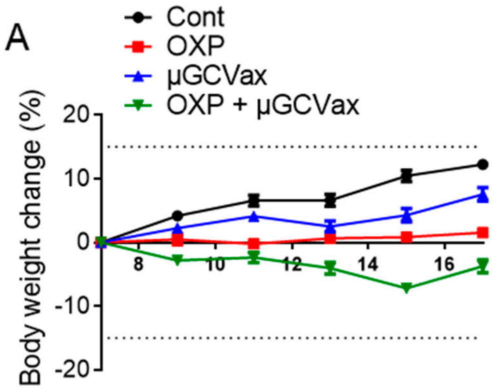

Figure S2. The relative body weight change of CT26 tumor-bearing mice after treatments. (**A**) The relative body weight change of CT26 tumor-bearing mice after treatments. n = 5 mice per group. Cont, control; OXP, oxaliplatin.

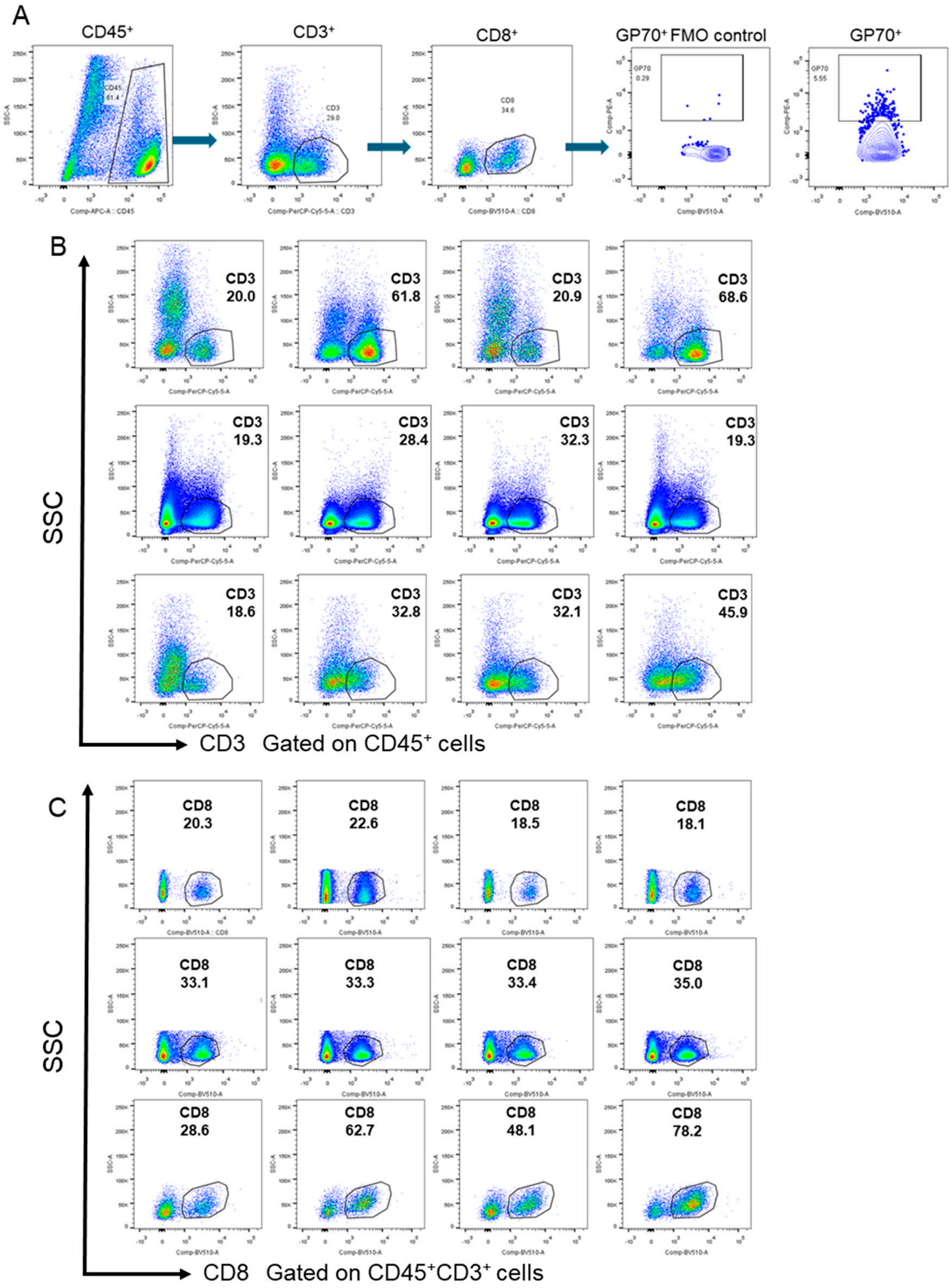

Figure S3. Combination of oxaliplatin and μGCVax enhances T cell infiltration and increases tumor-specific CD8⁺ T cells. (**A**) Representative gating strategy for the T cells and GP70⁺ CD8 T cells in tumor. **(B)** Representative flow cytometry plots of CD3⁺ T cells in blood, spleen and tumor after 17 days of tumor inoculation. Cells in the CD45⁺ gate are shown. (**C**) Representative flow cytometry plots of CD8⁺ T cells in blood, spleen and tumor. Cells in the CD45⁺CD3⁺ gate are shown. n = 5 mice per group. Cont, control; OXP, oxaliplatin; CD3TC, CD3⁺ T cells.

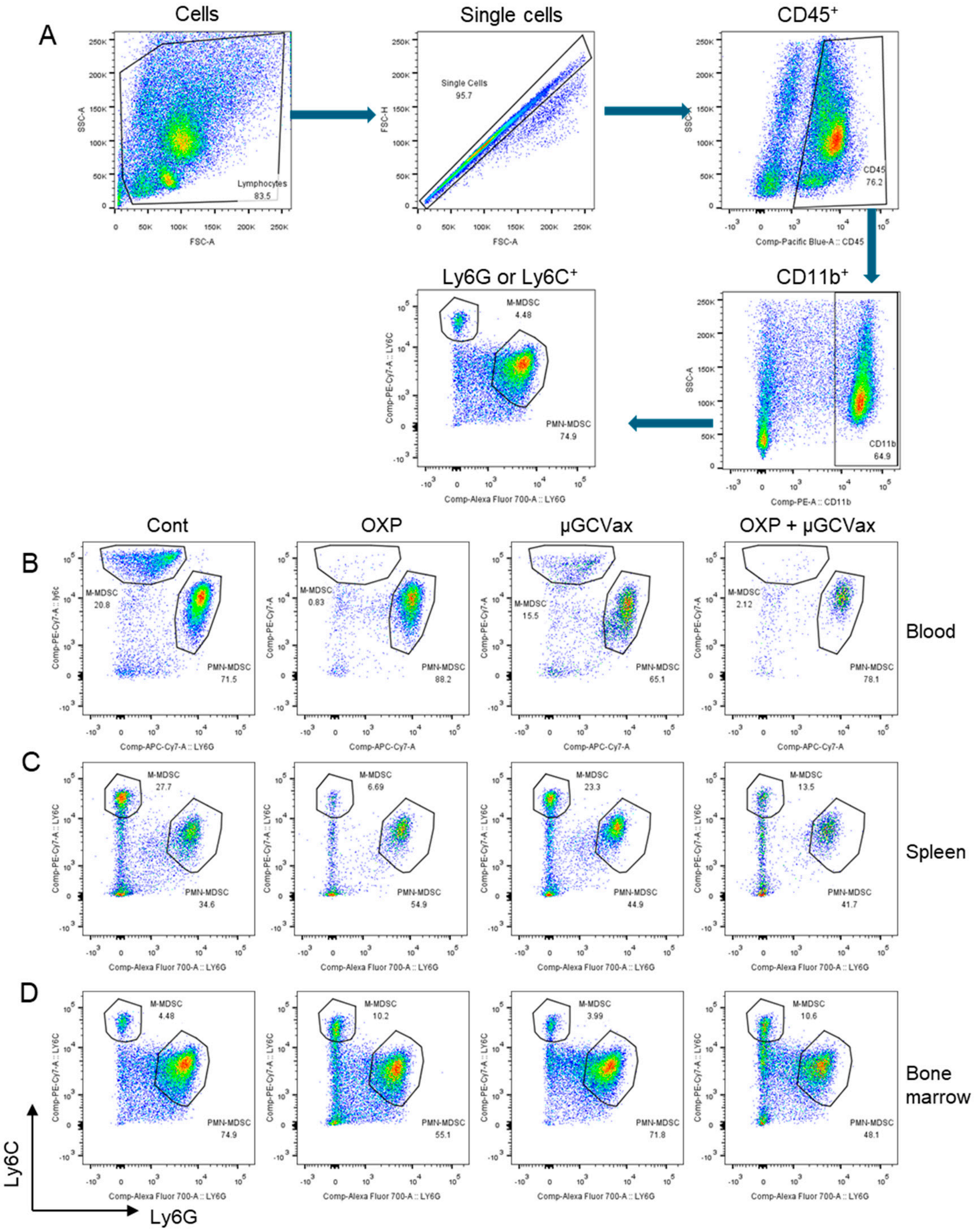

Figure S4. Oxaliplatin depleted MDSCs in CT26 tumor-bearing mice. (A) Representative gating strategy for the M-MDSC and PMN-MDSC in bone marrow. (**B-D**) Representative flow cytometry plots of MDSCs in blood, spleen and bone marrow after 17 days of tumor inoculation. Cells in the $CD45^{+}CD11b^{+}$ cell population gate are shown. M-MDSCs (identified as $CD45^{+}CD11b^{+}Ly6C^{+}$), PMN-MDSCs (identified as $CD45^{+}CD11b^{+}Ly6G^{+}$), T-MDSCs (Total MDSCs, M-MDSCs plus PMN-MDSCs). n = 5 mice per group. Cont, control; OXP, oxaliplatin.

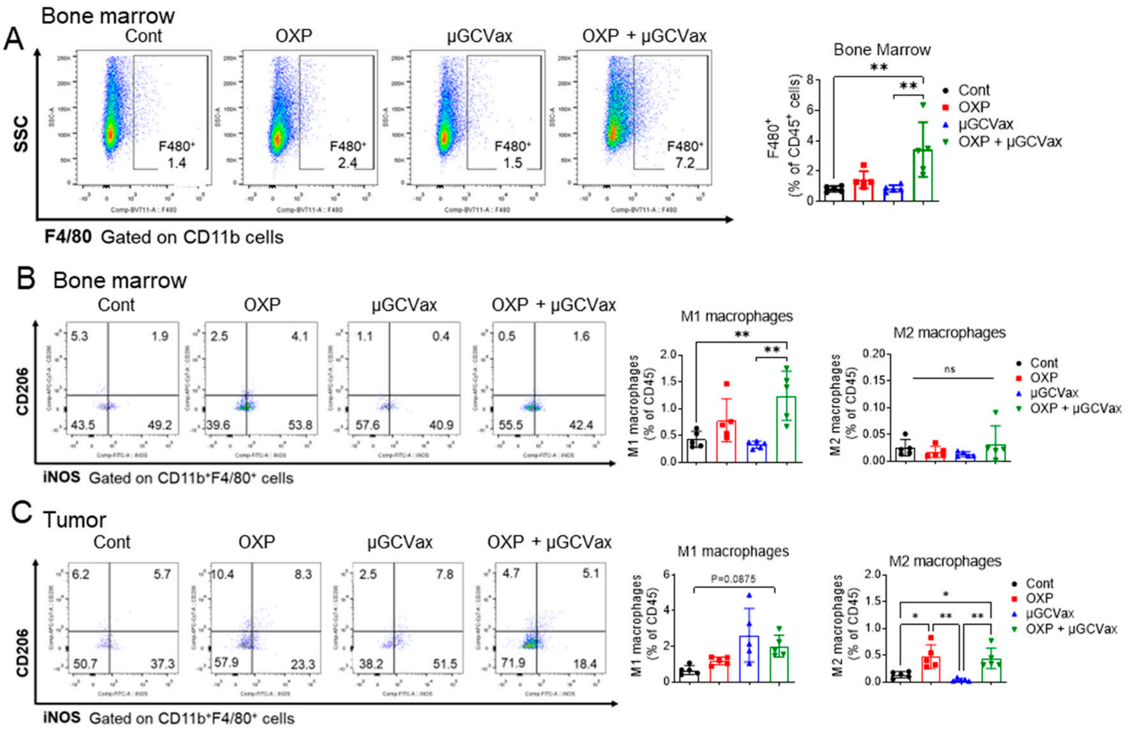

Figure S5. Oxaliplatin modulated macrophage polarization. (**A**) The proportions of F4/80⁺ cells in CD45⁺CD11b⁺ cells in bone marrow. **(B-C)** Representative flowcytometry plots of macrophages in the bone marrow (B) and tumor (C) 17 days after tumor inoculation. Cells within the CD11b⁺F4/80⁺ gate are shown. M1 macrophages identified as CD45⁺CD11b⁺F4/80⁺ iNOS⁺CD206⁻, M2 macrophages identified as CD45⁺CD11b⁺F4/80⁺ iNOS⁻CD206⁺. n = 5 mice per group. Cont, control; OXP, oxaliplatin. One-way ANOVA followed by Tukey's multiple comparisons test for panels A-C. ns, not significant, $^{*}p < 0.05$, $^{**}p < 0.01$.

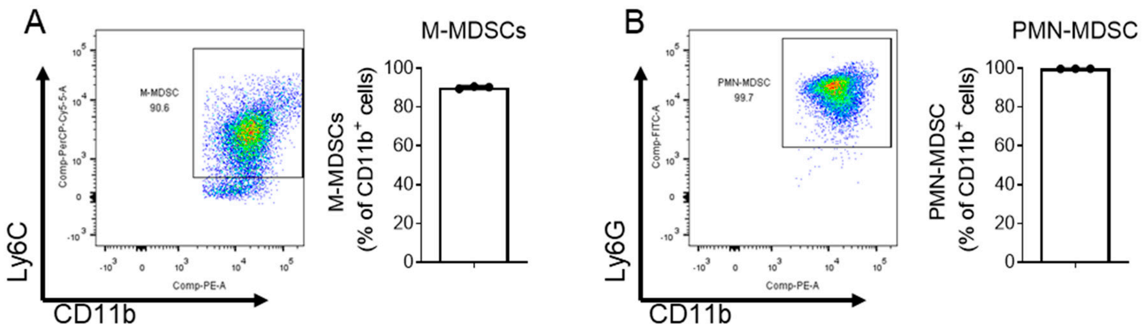

Figure S6. Purity of isolated M-MDSCs and PMN-MDSCs. M-MDSCs and PMN-MDSCs were purified from bone marrow of CT26-bearing mice went through treatments. The proportion of M-MDSCs (identified as $CD45^+CD11b^+Ly6C^+$) in $CD11b^+$ cells (**A**) and PMN-MDSCs (identified as $CD45^+CD11b^+Ly6G^+$) in $CD11b^+$ cells (**B**) were examined by flow cytometry. n = 3 mice per group.

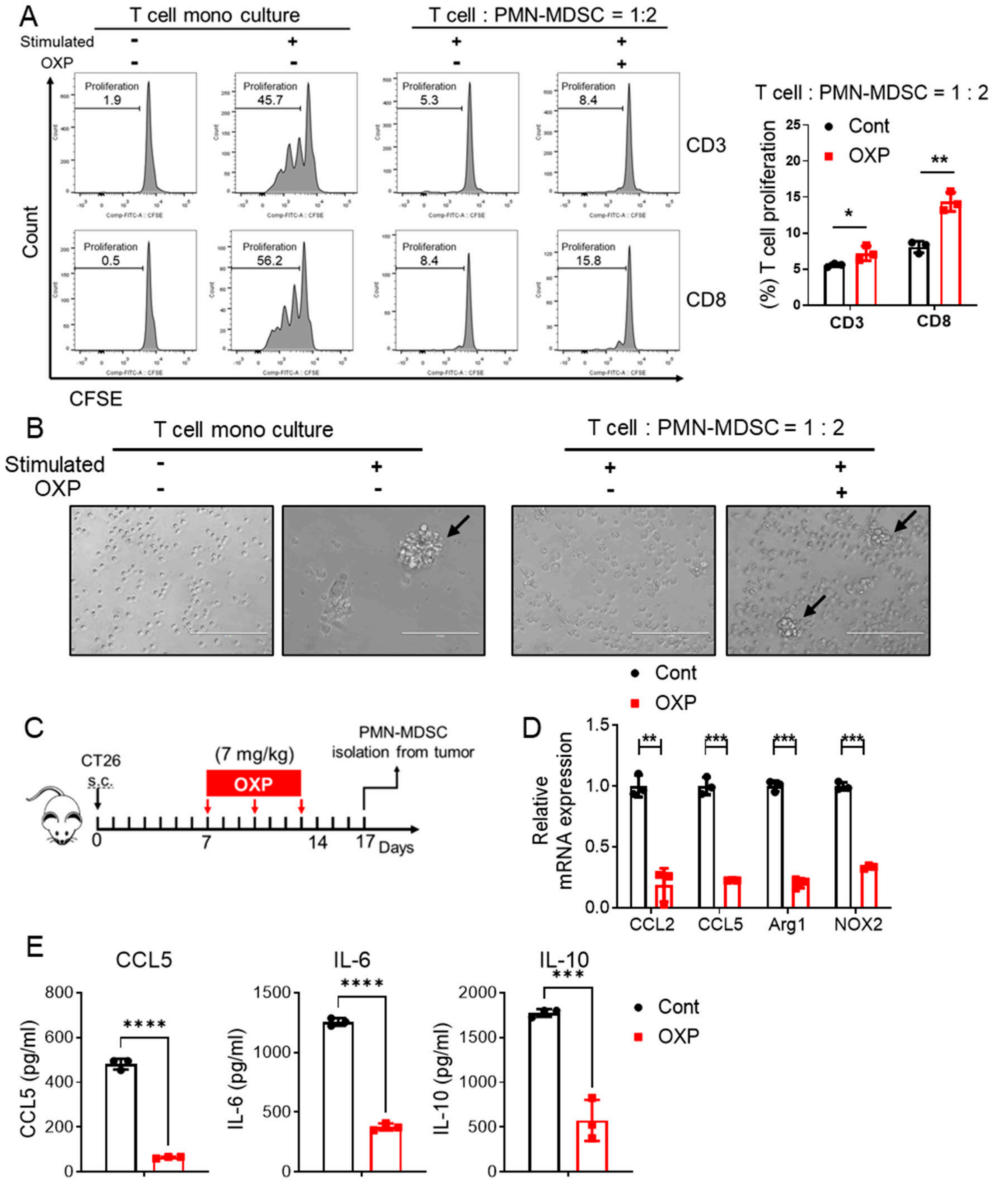

Figure S7. Oxaliplatin suppressed the function of PMN-MDSCs *in vivo* and *in vitro*. (**A**) (**A**) Bone marrow-derived PMN-MDSCs were pre-treated with oxaliplatin (5 μg/mL) for 20 h and co-cultured with CFSE-labeled splenic T cells (1:2) for 72 h with PMA (10 ng/mL) and ionomycin (500 ng/mL). T cell proliferation was analyzed by flow cytometry. **(B)** Bone marrow–derived PMN-MDSCs were pre-treated with oxaliplatin (5 μg/mL) for 20 h and co-cultured with CFSE-labeled splenic T cells (1:2) for 72 h in the presence of PMA (10 ng/mL) and ionomycin (500 ng/mL). T cell clusters were visualized by microscopy. Scale bar, 100 μm. **(C)** Treatment scheme. Mice were injected with 1 × 10^6 CT26 cells and given oxaliplatin on days 7, 10, and 13. PMN-MDSCs were isolated on day 17. (**D**) Real-time quantitative PCR was performed to examine the mRNA level of CCL2, CCL5, Arg1 and NOX2 of Tumor-infiltrating PMN-MDSCs from Cont or OXP-treated mice. (**E**) Tumor-infiltrating PMN-

MDSCs from Cont or OXP treated mice were cultured for 24 h. The concentrations of CCL5, IL6 and IL10 in the supernatant were detected by ELISA. For each group samples were prepared in triplicate. Cont, control; OXP, oxaliplatin. Two-tailed Student's t test for panels A, D and E. ns, not significant. *p < 0.05, **p < 0.01, ***P < 0.001, ****p < 0.0001.
